# Targeting CXCR4 potentiates anti-PD-1 efficacy modifying the tumor microenvironment and inhibiting neoplastic PD-1

**DOI:** 10.1186/s13046-019-1420-8

**Published:** 2019-10-28

**Authors:** Crescenzo D’Alterio, Maria Buoncervello, Caterina Ieranò, Maria Napolitano, Luigi Portella, Giuseppina Rea, Antonio Barbieri, Antonio Luciano, Giosuè Scognamiglio, Fabiana Tatangelo, Anna Maria Anniciello, Mario Monaco, Ernesta Cavalcanti, Piera Maiolino, Giulia Romagnoli, Claudio Arra, Gerardo Botti, Lucia Gabriele, Stefania Scala

**Affiliations:** 1 Functional Genomics, Istituto Nazionale Tumori “Fondazione G. Pascale”, IRCCS, 80,131 Naples, Italy; 2 Research Coordination and Support Service, Istituto Superiore di Sanità, Viale Regina Elena 299, 00161 Rome, Italy; 3 Animal Facility, Istituto Nazionale Tumori “Fondazione G. Pascale”, IRCCS, 80,131 Naples, Italy; 4 Pathology, Istituto Nazionale Tumori “Fondazione G. Pascale”, IRCCS, 80,131 Naples, Italy; 5 Division of Laboratory Medicine, Department of Pathology and Laboratory Diagnostics, Istituto Nazionale Tumori “Fondazione G. Pascale”, IRCCS, 80,131 Naples, Italy; 6 Pharmacy, Istituto Nazionale Tumori “Fondazione G. Pascale”, IRCCS, 80,131 Naples, Italy; 7 Department of Haematology, Oncology and Molecular Biology Istituto Superiore di Sanità, Viale Regina Elena, 299, 00161 Rome, Italy

**Keywords:** Tumor microenvironment, Immune privilege, Tumor infiltrating lymphocytes, Treg, MDSC; CXCR4-CXCL12 pathway, Tumor intrinsic PD-1 pathway

## Abstract

**Background:**

Inefficient T-cell access to the tumor microenvironment (TME) is among the causes of tumor immune-resistance. Previous evidence demonstrated that targeting CXCR4 improves anti-PD-1/PD-L1 efficacy reshaping TME. To evaluate the role of newly developed CXCR4 antagonists (PCT/IB2011/000120/ EP2528936B1/US2013/0079292A1) in potentiating anti-PD-1 efficacy two syngeneic murine models, the MC38 colon cancer and the B16 melanoma-human CXCR4-transduced, were employed.

**Methods:**

Mice were subcutaneously injected with MC38 (1 × 10^6^) or B16-hCXCR4 (5 × 10^5^). After two weeks, tumors bearing mice were intraperitoneally (ip) treated with murine anti-PD-1 [RMP1–14] (5 mg/kg, twice week for 2 weeks), Pep R (2 mg/kg, 5 days per week for 2 weeks), or both agents. The TME was evaluated through immunohistochemistry and flow-cytometry. In addition, the effects of the human-anti-PD-1 nivolumab and/or Peptide-R54 (Pep R54), were evaluated on human melanoma PES43 cells and xenografts treated.

**Results:**

The combined treatment, Pep R plus anti-PD-1, reduced the MC38 Relative Tumor Volume (RTV) by 2.67 fold (*p* = 0.038) while nor anti-PD-1, neither Pep R significantly impacted on tumor growth. Significant higher number of Granzyme B (GZMB) positive cells was detected in MC38 tumors from mice treated with the combined treatment (*p* = 0.016) while anti-PD-1 determined a modest but significant increase of tumor-infiltrating GZMB positive cells (*p* = 0.035). Also, a lower number of FoxP3 positive cells was detected (*p* = 0.022). In the B16-hCXCR4 tumors, two weeks of combined treatment reduced tumor volume by 2.27 fold while nor anti-PD-1 neither Pep R significantly impacted on tumor growth. A significant higher number of GRZB positive cells was observed in B16-hCXCR4 tumors treated with combined treatment (p = 0,0015) as compared to anti-PD-1 (*p* = 0.028). The combined treatment reduced CXCR4, CXCL12 and PD-L1 expression in MC38 tumors. In addition, flow cytometry on fresh B16-hCXCR4 tumors showed significantly higher Tregs number following anti-PD-1 partially reversed by the combined treatment Pep R and anti-PD-1. Combined treatment determined an increase of CD8/Tregs and CD8/MDSC ratio. To dissect the effect of anti-PD-1 and CXCR4 targeting on PD-1 expressed by human cancer cells, PES43 human melanoma xenograft model was employed. In vitro human anti-PD-1 nivolumab or pembrolizumab (10 μM) reduced PES43 cells growth while nivolumab (10 μM) inhibited pERK1/2, P38 MAPK, pAKT and p4EBP. PES43 xenograft mice were treated with Pep R54, a newly developed Pep R derivative (AcHN-Arg-Ala-[DCys-Arg- Nal(2′)-His-Pen]- COOH), plus nivolumab. After 3 weeks of combined treatment a significant reduction in tumor growth was shown (*p* = 0.038). PES43 lung disseminated tumor cells (DTC) were detected in fresh lung tissues as melanoma positive MCSP-APC^+^ cells. Although not statistically significant, DTC-PES43 cells were reduced in mice lungs treated with combined treatment while nivolumab or Pep R54 did not affect DTC number.

**Conclusion:**

Combined treatment with the new developed CXCR4 antagonist, Pep R, plus anti-PD-1, reduced tumor-growth in two syngeneic murine models, anti-PD-1 sensitive and resistant, potentiating Granzyme and reducing Foxp3 cells infiltration. In addition, the human specific CXCR4 antagonist, Pep R54, cooperated with nivolumab in inhibiting the growth of the PD-1 expressing human PES43 melanoma xenograft. This evidence sheds light on PD-1 targeting mechanisms and paves the way for CXCR4/PD-1 targeting combination therapy.

## Background

Unprecedented rates of long-lasting tumor responses can be achieved in patients with a variety of cancers blocking the immune checkpoints with inhibitors (ICI) such as antibodies targeting cytotoxic T lymphocyte–associated protein 4 (CTLA-4) or the programmed cell death 1 (PD-1) pathway [1]. However durable responses occur in a minority of patients among which 25% eventually relapse [1]. Patients respond to ICI because of a pre-existing antitumor T cell response that retains therapeutic potential until the infiltrating T cells engage their T cell receptor (TCR), triggering expression of PD-1 on T cells, releasing IFNγ [2] with reactive expression of PD-L1 by cancer-resident cells [1]. Among reasons of tumor resistance there is an active T cell exclusion [3]. In addition, recent studies revealed intrinsic functional expression of PD-1 that contributes to tumor immunoresistance. In melanoma cells, PD-1 can be activated by its ligand PD-L1 expressed by tumor cells, modulating downstream mammalian target of rapamycin signaling and promoting tumor growth independent of adaptive immunity. In liver cancer cells, bladder cancer as well as in non-small lung cancer cells [4–7] intrinsic PD-1 signaling was reported. The chemokine receptor 4 (CXCR4), is an evolutionarily highly conserved GPCR expressed on monocytes, B cells, and naive T cells in the peripheral blood. Its ligand, CXCL12, is a homeostatic chemokine, which controls hematopoietic cell trafficking, adhesion, immune surveillance and development. CXCR4 is overexpressed in more than 23 human cancers and controls metastatic dissemination in the majority of tumors in which is overexpressed [8]. Targeting the CXCR4–CXCL12 axis exerts activity on the TME reverting the tolerogenic polarization of the TME rich of immunosuppressive cells such as regulatory T cells (Treg), M2, and N2 neutrophils [9–11]. Favoring spatial distribution of effector T cells, recruitment of tumor-specific T cells from the vessel and T cells proliferation, CXCR4 axis modulates ICI responsiveness [12]. CXCR4 antagonists potentiate ICI effect in HCC xenograft [9], in murine intraperitoneal papillary epithelial ovarian cancer [13] and in murine colorectal cancer where NOX-A12, the CXCL12 antagonist L-RNA-aptamer, enhanced CD8 and NK infiltration [14]. To target CXCR4 a new family of CXCR4 peptide antagonists was developed and Peptide R is the lead compound (Pep R) (H-Arg-Ala-[Cys-Arg-Phe-Phe-Cys]-CO2H) [15–18]. Pep R inhibits CXCR4 dependent cell migration and lung metastasis development [15]. Through a lead compound optimization process [19, 20] Peptide R54 (Pep R54), (AcHN-Arg-Ala-[DCys-Arg- Nal(2′)-His-Pen]- COOH) was developed displaying better serum stability and higher CXCR4 affinity than Pep R (IC50 = 20 ± 2 nM) [19].

Aim of the work was to evaluate Pep R in potentiating the anti–PD-1 efficacy in two syngeneic murine models, the colon cancer MC38 cells [21–23] and the B16 melanoma model [22, 24] –human CXCR4 transduced, respectively reported to be immune responsive [21–23] and immune resistant cancer models [22, 24]. Moreover, the effect of targeting tumoral intrinsic PD-1, as T cell-independent effect, plus CXCR4 antagonism was evaluated in PES43 human melanoma CXCR4 expressing [25] xenografts.

## Materials and methods

### Cell lines

MC38 murine colon cancer cells were kindly provided by Dr. Gabriele (Istituto Superiore di Sanità), cultured in Dulbecco’s Modified Eagle Medium (DMEM) containing 10% fetal bovine serum (FBS) and grown at 37 °C in 5% CO_2_. B16BL6/F10 murine melanoma cells were transfected with pYF1-fusin plasmid containing human CXCR4 gene (kindly provided by Dr. Aloj, NCI “Pascale”, Naples, Italy) according to FuGEN 6 protocol (Roche Applied Science, Indianapolis, IN). The B16-human-CXCR4 cells were grown in Iscove’s Modified Dulbecco’s Medium (IMDM with 10% FBS) plus 100 μg/mL G418 [15]. PES43 human melanoma cancer cells, [25] were cultivated in IMDM.

### In vivo studies

1. C57Bl/6 mice were subcutaneously inoculated with MC38 (1 × 10^6^) murine colon cancer cells. When mean tumor volume reached approximately 250 mm^3^, treatment started (typically 8 mm × 8 mm tumor, after ~ 20 days post-injection) [26]. Treatment was conducted intraperitoneally (ip) as follow: Peptide R (2 mg/kg) (GL Biochem Shangay LTD) ip, 5 days week/2 weeks; Rat IgG2a, anti-mouse PD-1 (CD279) monoclonal antibody (RMP1–14, BioXCell), 5 mg/kg ip, twice a week/2 weeks (Additional file 1: Figure S1A). For the combined treatment, Pep R was inoculated 1 h before anti-PD-1 to avoid cross*-*reactivity and to administrate anti-PD-1 in a CXCR4-inhibited TME context [20]. Tumor volume was calculated using the formula: V = (L × W^2^)/2, where L and W are the long and short diameters of the tumor, respectively. Relative Tumor volume (RTV) is the ratio between Vx caliper-derived volumes in mm^3^ at a given time and V1 at the start of treatment (RTV = Vx/V1) [27]. RTV mean ± SEM from caliper-measured data was evaluated for each mouse (*n* = 4 per group) every other day.
2. C57Bl/6 mice were subcutaneously injected with B16-hCXCR4 (5 × 10^5^) cells and treated as above. Treatment started when tumor masses become palpable (approximately 10 days post-injection). Mean tumor volumes from caliper-measured data were evaluated for each mouse, (*n* = 8–12 per group) every other day. Tumor length and width were measured using a digital caliper. Tumor volume was estimated with the formula: (L × W^2^)/2.
3. Athymic Nude-Foxn1nu mice were subcutaneously injected with PES43 (2.5 × 10^6^) melanoma cells. Treatment started when tumor mass was ~ 50 mm^3^. Treatment was conducted intraperitoneally with Peptide R54 (GL BiochemShangay LTD) (2 mg/kg ip, 5 days week/3 weeks), fully anti-human PD-1/(CD279) IgG4 (S228P) (nivolumab) monoclonal antibody (5 mg/kg ip, twice week/3 weeks) (Additional file 1: Figure S1B). Tumor length and width were measured using a digital caliper. Tumor volume was estimated with the formula: (L × W^2^)/2. Mean tumor volumes were evaluated for each mouse, (n = 8–9 per group) 3 times/week. Animal studies were performed in compliance with the ARRIVE guidelines and with the principle of the “3Rs” (Replacement, Reduction and Refinement). Italian Ministry of Health permission 10,047/2017PR 13/02/2017) (Italian decree n. 26 04/03/2014 /European Directive 2010/63/EU). A priori power analysis was conducted using the Gpower program (G*Power software package, version 3.1.4). All mice were monitored every other day for body weight, sign hydration (skin tents), posture, grooming (hunched posture, ruffled fur), and activity (animal do not move, moves only when touched, abnormal gait). No sign of toxicity was reported with either treatment in the three models.

#### Immunohistochemistry (IHC)

Paraffin-embedded sections (3 μm) were dewaxed and rehydrated, antigen retrieval was performed by Decloaking Chamber™ NxGen (Biocare Medicals) designed for heat-induced epitope retrieval (HIER) with Antigen Unmasking Solution (pH 6). After blocking with the appropriate serum designed for blocking endogenous mouse IgG and non-specific background in mouse tissues (Rodent Block M; Biocare Medical), samples were incubated overnight at 4 °C using primary antibodies: FoxP3 (ab50501; 1:500 diluted; 1 h room temperature incubation); Granzyme B GZMB (ab4059; 1:300 diluted; 1 h room temperature incubation); CXCR4 (NB100–74396; 1:200 diluted; overnight + 4 °C incubation) CXCR7 (ab38089; 1:100 diluted; overnight + 4 °C incubation); CXCL12 (Human/Mouse CXCL12/SDF-1 mouse mAb Clone # 79018; Novus Biologicals 1:50; diluted; overnight + 4 °C incubation); PD-1 (#PA5–20350 Rabbit pAb Invitrogen™ 1:50 diluted; overnight + 4 °C incubation) PD-L1 (17952–1-AP, 1:50 Rabbit pAb, Proteintech Group, Inc. and validated with PD-L1 (E1L3N®) XP® Rabbit mAb #13684 Cell Signaling Technology, Inc.1:200; diluted; overnight + 4 °C incubation). Ki67 (M7240 Dako 1:75 diluted; overnight + 4 C incubation). Immune cells were evaluated from the invasive margin to tumor core in at least 3–4 regions of interest for each slide at low power (100× magnification) and cells counted in 5 consecutive not overlapping high-power field (HPF) 400x magnification (0.237 mm^2^ / field), using an Olympus BX51 microscope (Olympus, Tokyo, Japan). The evaluation of stained immune cells was conducted in duplicate by three independent trained observers (FT; CD and GS). The results were expressed as mean of positively stained immune cells/mm^2^.

#### Flow cytometry

For fluorescence-activated cell sorting (FACS) analysis, B16-hCXCR4 melanoma explants were cut into small fragments using curved scissors and then digested in type III collagenase-containing medium (7 mg/ml; Worthington) and DNase I (2 mg/ml; Worthington) for 30 min at room temperature in agitation, followed by EDTA (0.1 M, pH 7.2) for an additional 5 min. The homogenate was then passed through a cell strainer and cells were incubated with FcR Block (Miltenyi Biotic) as indicated by the manufacturer at 4 °C. Surface staining was performed in the dark for 30 min at 4 °C in staining buffer. Cells were washed and stained with a viability dye (eFluorTM780, eBioscience) prior to fixation procedures with 2% paraformaldehyde. Cells were then divided into five different staining groups to sub-gate: dendritic cells, granulocyte and monocyte/macrophage subsets, lymphocytes, Treg cells. Dendritic cell antibody cocktail: Brilliant Violet 510 (BV510) CD45 (BD Pharmingen),phycoerythrin (PE) CD11c (BD Pharmingen), fluorescein isothiocyanate (FITC) anti-CD103 (Miltenyi), allophycocyanin (APC) CD11b (eBioscience), biotin PDCA1 (Miltenyi). Granulocyte and monocyte/macrophage subsets antibody cocktail: BV510 anti-CD45, FITC anti-Ly6G (BD Pharmingen), PE anti-CD11c, biotin F4/80 (Caltag), PE-Cy7 anti-IA/IE (Thermo Fisher), APC anti-CD11b, Pacific Blue (PB) anti-Ly6C (Biolegend). Treg cells: BV510 anti-CD45, FITC anti-CD4 (eBioscience), APC anti-CD25 (BD Pharmingen), PE anti-FoxP3 (Biolegend). Biotynilated antibodies were detected by SteptavidinPerCP5.5 or PB (BD Pharmingen). For intracellular staining of PE anti-FoxP3, the manufacturer protocol was followed (eBioscience Intracellular Fixation & Permeabilization Buffer Set). Flow cytometry was performed on a Gallios flow cytometer and analysed with the Kaluza Analysis Software (Beckman Coulter).

#### Disseminated tumoral cells (DTCs)

Murine lungs were cut into small fragments using curved scissors. The homogenate was processed through a cell strainer and cells were incubated with FcR Block at 4 °C. Surface staining was performed with Anti-human Melanoma MCSP-APC that identify the melanoma-associated chondroitin sulfate proteoglycan (MCSP) antigen (Miltenyi Biotec) in the dark for 30 min at 4 °C.

#### Immunoblotting

Cells were lysed in a whole-cell buffer containing protease and phosphatase (10 mM NaF, 10 mM Na-pyrophosphate, 1 mM Na3VO4) inhibitors. Rabbit monoclonal antibodies for p44/42 MAPK (Erk1/2), phospho-p44/42 MAPK (Erk1/2; T202/Y204), anti-4EBP1, anti-phospho 4EBP1, phosphor-P38 MAPK (T180/Y182), P38 MAPK, Akt and phospho-Akt (phospho-Ser-473) antibodies were from Cell Signaling (Danvers, MA, USA). Secondary antibodies include goat anti-rabbit-HRP (Jackson ImmunoResearch, West Grove, PA, USA) and goat anti-mouse-HRP (Santa Cruz Biotechnology).

### Lactate assay

Quantitative determination of lactate in plasma from retro-orbital bleeding was assessed by Cobas C Analyzer (Lactate Gen. 2 – Roche Diagnostics).

### Statistical analysis

SPSS software (version 13.0) and MedCalc (version 12.3.0) were used for statistical analysis. Data were expressed as the mean ± SEM or ± SD as stated in figure legends. For multiple groups comparison, Repeated Measures ANOVA (RMANOVA) with Tukey HSD post hoc-test was used to determine treatment effect over time. The continuous variables were compared using an unpaired Student t test or a Mann-Whitney U test if the variables were not normally distributed. For multiple groups comparison was used one way ANOVA or Kruskal-Wallis test if the variables were not normally distributed. *P* < 0.05 was considered to indicate a statistically significant difference.

## Results

### The CXCR4 antagonist Pep R potentiates anti-PD-1 efficacy in murine MC38 colon cancer and B16-hCXCR4 melanoma

**Fig. 1:**
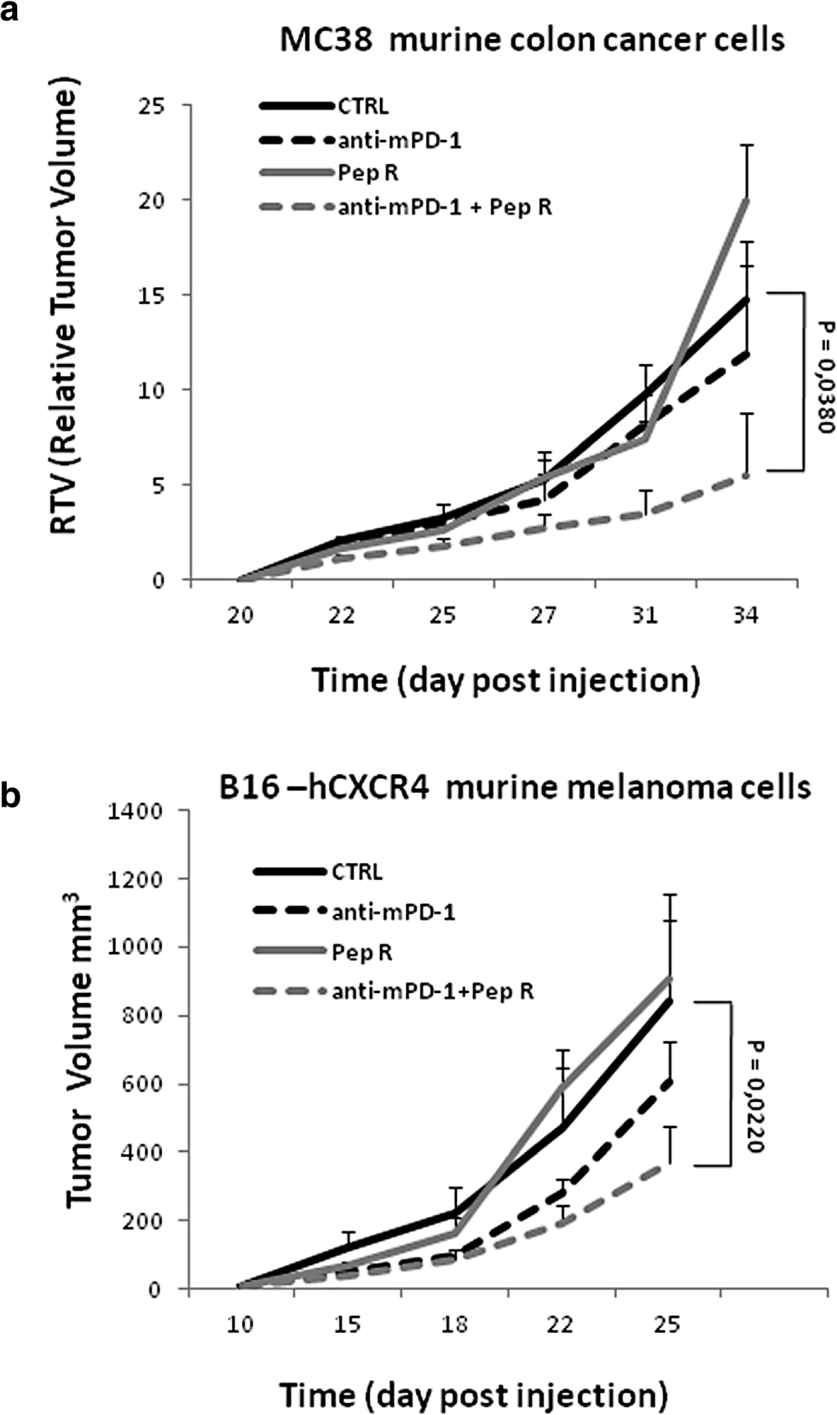
Pep R potentiates the anti-PD-1 antitumor efficacy in murine MC38 colon cancer and B16-human-CXCR4 mice models. **a**. MC38 colon cancer. Mice were subcutaneously inoculated with 1 × 10^6^ MC38 colon cancer cells. When tumors reached 250 mm^3^ volume (day 20) mice were randomized and treated for 2 weeks according to treatment schedule (Peptide R 2 mg/kg ip, 5 day/week; anti–PD-1 5 mg/kg i.p. twice weekly). Relative Tumor Volume (RTV) (mean ± SEM, *n* = 4 per group), untreated (n = 4), anti-mouse PD-1 (n = 4), Pep R (n = 4), anti-PD-1 + Pep R combination (n = 4). RTV: Untreated 14.75 ± 3.07; anti-PD-1 11.91 ± 4.60, Pep R 20.0 ± 2.95; anti-PD-1 + Pep R 5.52 ± 3.22. **b**. B16 melanoma-human-CXCR4. Mice were subcutaneously inoculated with 5 × 10^5^ B16-human-CXCR4 cells and treated as above. Mean tumor volumes (MTV) ± SEM. Untreated mice (*n* = 12), anti-PD-1 (*n* = 10), Pep R (*n* = 8), anti-PD-1 + Pep R combination (n = 10). Response time trends recorded for different treatment was analyzed by comparing means at each time point using repeated measures analysis of variance (RMANOVA with Tukey HSD post hoc-test);* *p* < 0.05

To evaluate the effect of CXCR4 antagonist Pep R in modulating anti-PD-1 efficacy two syngeneic mice tumor models were employed. MC38 murine colon cancer [28] were previously characterized as responders to anti-PD-1 therapies [21], and murine melanoma B16-hCXCR4 previously defined as poor immune responsive tumors [22, 24, 29]. C57Bl/6 mice were subcutaneously inoculated with MC38 (1 × 10^6^) murine colon cancer cells [26, 28, 30, 31]. Treatment was conducted intraperitoneally (ip) as follow: Peptide R (2 mg/kg) ip, 5 days week/2 weeks; anti-mouse PD-1 (CD279) monoclonal antibody, 5 mg/kg ip, twice a week/2 weeks (Additional file 1: Figure S1A). For combined treatment, Pep R was inoculated 1 h before anti-PD-1 to prevent cross*-*reactivity. Treatment lasted two weeks reported to be sufficient to assess change in tumor growth [9, 14, 32]. The combined treatment with anti-PD-1 + Pep R reduced the MC38 Relative Tumor Volume (RTV) by 2.67 fold as compared to untreated tumors (*p* = 0.038; 95% CI: 1374–5,44 RMANOVA with Tukey HSD post hoc-test) (Fig. 1a) while nor anti-PD-1, neither Pep R significantly impacted on tumor growth.

B16-hCXCR4 (5 × 10^5^) cells were subcutaneously inoculated and treatment started when tumor masses become palpable (approximately 10 days post injection) [22, 24, 29]. Two weeks of combined treatment reduced tumor volume by 2.27 fold while nor anti-PD-1, neither Pep R significantly impacted on tumor growth (Fig. 1b).

### Targeting PD-1 and CXCR4 modifies the tumor microenvironment (TME) in MC38 and B16-hCXCR4 tumors

**Fig. 2:**
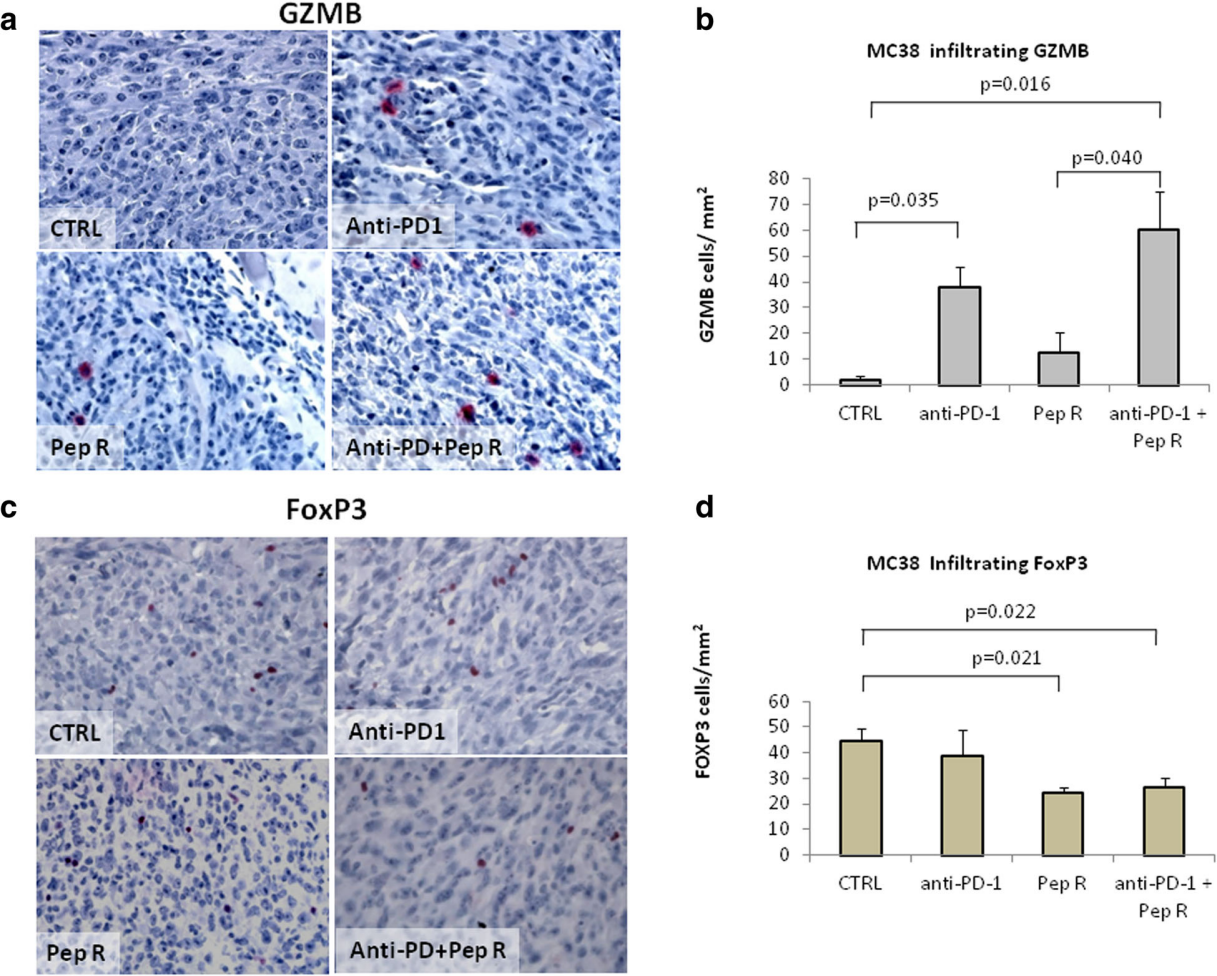
Pep R in combination with anti-PD-1 increased Granzyme B infiltration and reduced Treg recruitment in MC38 tumors. Granzyme B infiltration and FoxP3 positive immune cells was assessed by immunohistochemistry in tumors from MC38 (**a**-**d**). **a**. Representative microphotographs of Granzyme B across treatment groups. Granzyme B staining was mainly detected in cytoplasmic granules of cytolytic T-lymphocytes and natural killer cells (red staining). Counterstaining of nuclei by hematoxilyn (blue) (magnification 400x). **b**. GZMB was quantified with the AxioVision Imaging System version 4.8 microscope expressed as IHC positive cell/mm^2^. Untreated mice: = 2 ± 1.73 (*n* = 4); anti-PD-1 = 37.66 ± 8.38 (n = 4); Pep R = 12.33 ± 8.22 (n = 4); anti-PD-1 + Pep R = 60.33 ± 14.54 (n = 4);. **c**. Representative microphotographs of FoxP3. FoxP3 staining showed nuclear immunoreactivity in lymphocytes (red staining, magnification 400x). **d**. Quantification FoxP3 was expressed as IHC positive cell/mm^2^. Untreated mice: = 44.88 ± 4.46 (n = 4); anti-PD-1 = 38.66 ± 10.15 (n = 4); Pep R = 24.27 ± 3.85 (n = 4); anti-PD-1 + Pep R = 26.41 ± 2.21 (n = 4). ANOVA Tukey HSD Posthoc Test). Data are shown as mean ± SEM. ANOVA Posthoc Tukey HSD. * *P* < 0.05

**Fig. 3:**
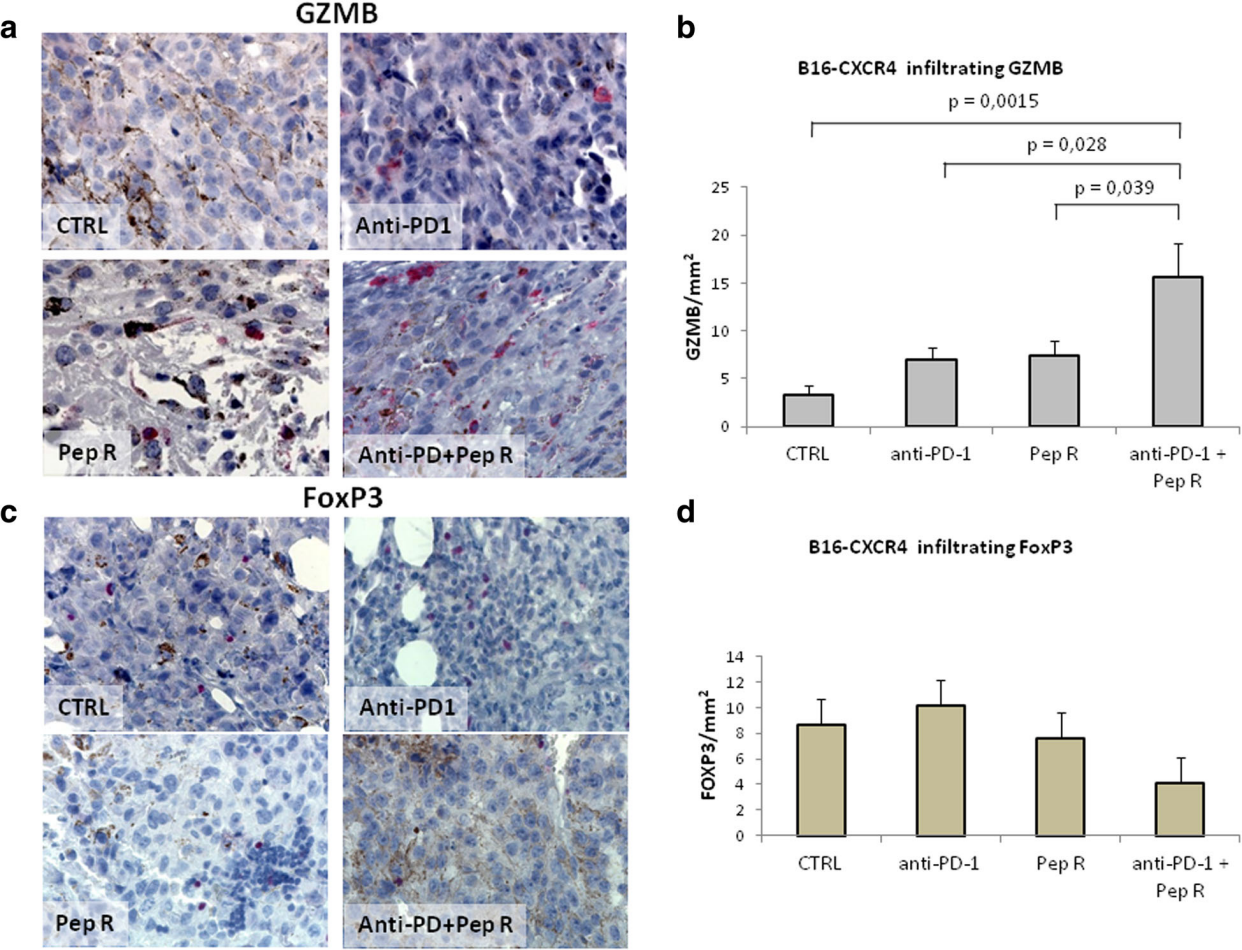
Pep R in combination with anti-PD-1 increased Granzyme B infiltration and reduced Treg recruitment in B16-hCXCR4 tumors. Granzyme B infiltration and FoxP3 positive immune cells was assessed by immunohistochemistry in tumors from B16-hCXCR4 (**a**-**d**). **a** Representative microphotographs of Granzyme B across treatment groups. Granzyme B staining was mainly detected in cytoplasmic granules of cytolytic T-lymphocytes and natural killer cells (red staining). Counterstaining of nuclei by hematoxilyn (blue) (magnification 400x). **b**. GZMB was quantified with the AxioVision Imaging System version 4.8 microscope expressed as IHC positive cell/mm^2^. Untreated mice: 3.23 ± 1.01 (n = 8); anti-PD-1 = 7.02 ± 1.19 (n = 8); Pep R = 7.44 ± 1.52 (n = 8); anti-PD-1 + Pep R = 15.6 ± 3.60 (*n* = 7). **c** Representative microphotographs of FoxP3. FoxP3 staining showed nuclear immunoreactivity in lymphocytes (red staining, magnification 400x). **d** Quantification FoxP3 was expressed as IHC positive cell/mm^2^. Data are shown as mean ± SEM. Untreated mice: 8.70 ± 2.17 (*n* = 5); anti-PD-1 = 10.18 ± 0.79 (*n* = 6); Pep R = 7.65 ± 3.31 (n = 8); anti-PD-1 + Pep R = 4.08 ± 1.70 (n = 6). ANOVA Posthoc Tukey HSD. * P < 0.05

We hypothesized that Pep R improved anti-PD-1 efficacy modifying the TME T*-*cell infiltration. Since Granzyme B positive staining represents a favorable indicator of antitumor activity [33]. Granzyme B (GRZB) positive immune cells were evaluated in the whole tumor sections. In Fig. 2a-b MC38 tumors from mice treated with combined treatment displayed a significant higher number of GZMB positive cells (*p* = 0.016) while, anti-PD-1 determined a modest but significant increase of tumor infiltrating GZMB positive cells (*p* = 0.035). As immunosuppressive cell infiltration impairs efficient immune response, Tregs cells were evaluated through FoxP3 immunostaining (Fig. 2c-d). Previous evidence demonstrated that CXCR4 is expressed on Tregs and that CXCR4 antagonism impairs patients derived Treg function [34]. A lower number of FoxP3 positive cells was detected in MC38 tumors treated with Pep R plus anti PD-1 (*p* = 0.022) (Fig. 2d). A significant higher number of GRZB positive cells was also observed in B16-hCXCR4 tumors treated with combination (p = 0,0015) as well as compared to anti-PD-1 (*p* = 0.028) or Pep R (*p* = 0.039) (Fig. 3a-b). Combined treatment trend towards a reduction of FoxP3 cell infiltration in B16-hCXCR4 tumors in (Fig. 3c-d).

**Fig. 4:**
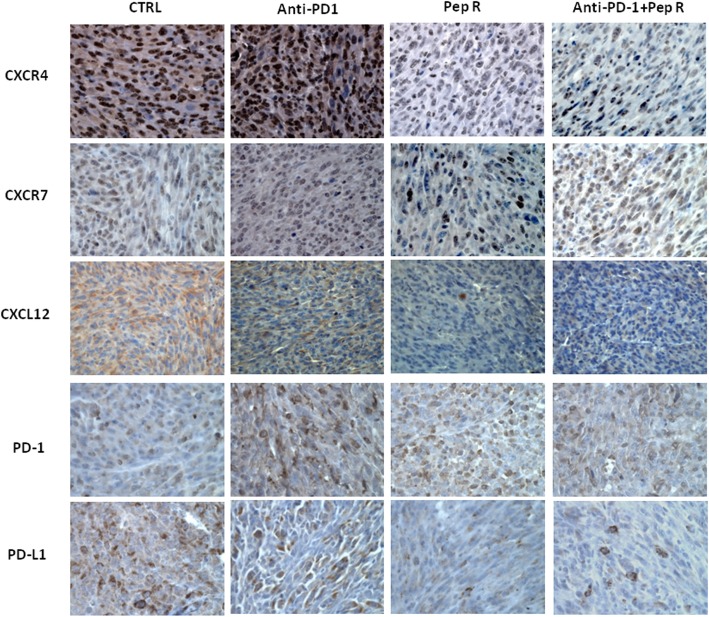
Pep R in combination with anti-PD-1 reduced the expression of CXCR4-CXCL12 and PD-L1 in MC38 tumors. Representative IHC pictures for CXCR4, CXCR7, CXCL12, PD-1 and PD-L1 expression (brown staining) in MC38 collected tumors (magnification 400x), from mice treated with Pep R, anti-murine PD-1 or combined treatment showing the reduction of CXCR4, CXCL12 and PD-L1 expression in mice treated with Pep R alone and in combination with anti-PD-1

**Fig. 5:**
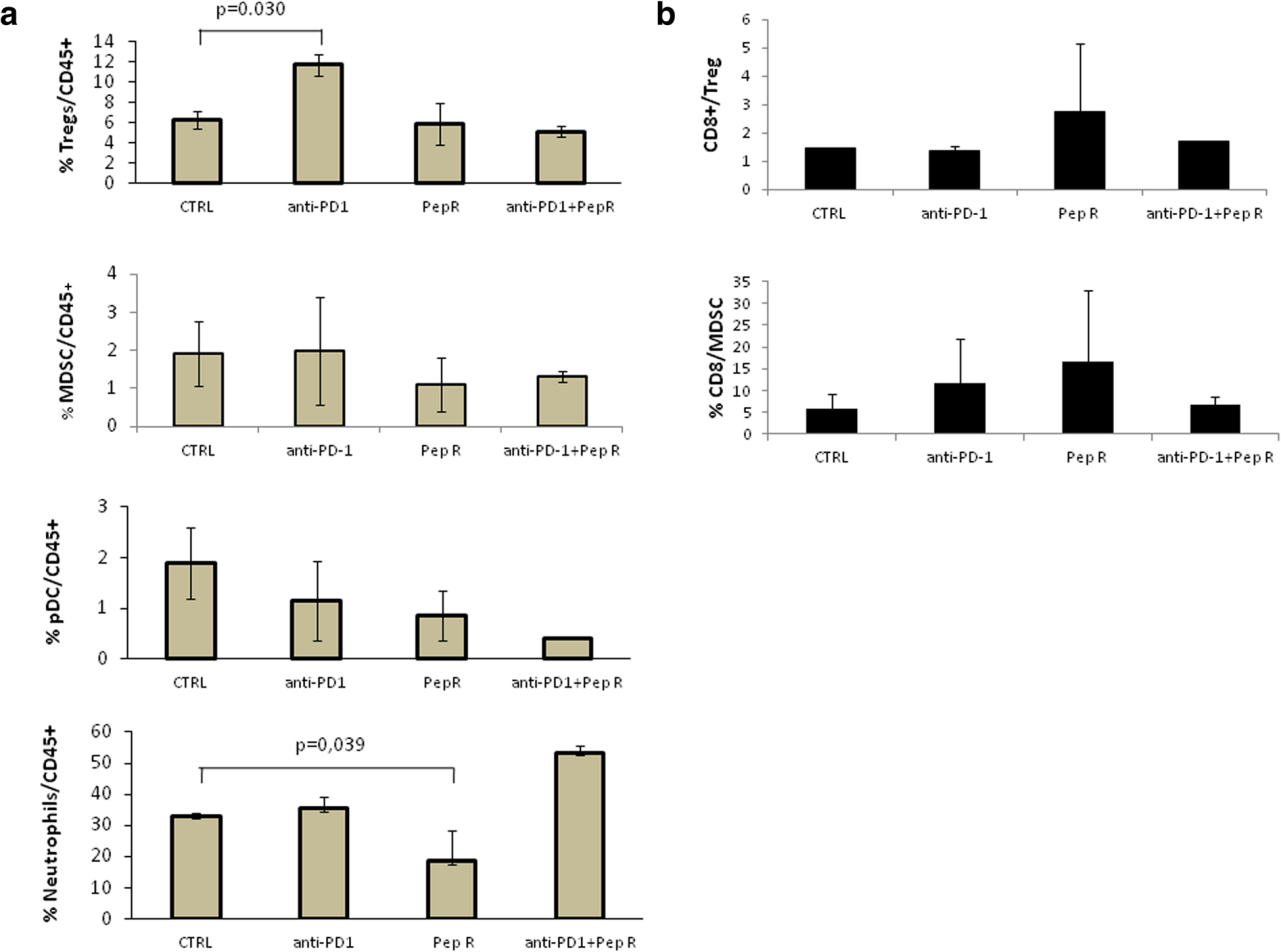
Pep R in combination with anti-PD-1 treatment mediated the impairment of infiltrating immune-suppressive cells in B16-hCXCR4 tumors. Flow cytometry analysis of single-cell suspensions from digested B16-hCXCR4 tumors (n = 6/group) stained as described in Material and Methods. The histograms represent the frequencies (mean ± SEM) of %: (**a**) Tregs/CD45+; MDSCs/CD45+; pDC/CD45+; neutrophil/CD45+; (**b**) CD8+/Treg, and CD8+/MDSCs; Mann-Whitney U test P < 0.05

CXCR4-CXCL12-CXCR7 expression was evaluated through immunohistochemistry in MC38 tumors. As shown in Fig. 4, CXCR4 decreased in tumors treated with Pep R or combined treatment while no variations were detected for the related receptor CXCR7, as expected due to CXCR4-Pep R specificity [15]. Interestingly a concomitant reduction of the ligand CXCL12 was reported. PD-1 expression was not affected by treatments in MC38 tumors, while PD-L1 reduction was revealed in both, anti-PD-1 and anti-PD-1 + Pep R treatments (Fig. 4). Thus, the combined treatment reduced CXCR4, CXCL12 and PD-L1 in MC38 TME confirming our previous evidence of Pep R specificity for CXCR4 [15–20, 34]. In addition, flow cytometry was conducted on fresh B16-hCXCR4 tumors. To evaluate TME treatment induced changes tumor-infiltrating Tregs, MDSC, pDC and Neutrophils were analyzed (Fig. 5a). In Fig. 5a a significant Tregs increase was detected in B16-hCXCR4 tumors anti-PD-1 treated, partially reversed by the combined treatment. A significant decrease in neutrophils infiltration was revealed in B16-hCXCR4 tumors treated with Pep R. Although not significant it is worth noting that pDC decreased with Pep R, anti-PD-1 and combined treatment (Fig. 5a). In Fig. 5b the ratio of CD8/Tregs and CD8/MDSC expressed modifications of effector/suppressor treatment-induced. The ratio CD8+ T cells/Tregs was higher in mice receiving Pep R, as a single treatment or in combination with anti-PD-1 [35] (Fig. 5b).

### Targeting CXCR4 potentiates nivolumab efficacy in PES43 human melanoma xenograft PD-1 expressing

**Fig. 6:**
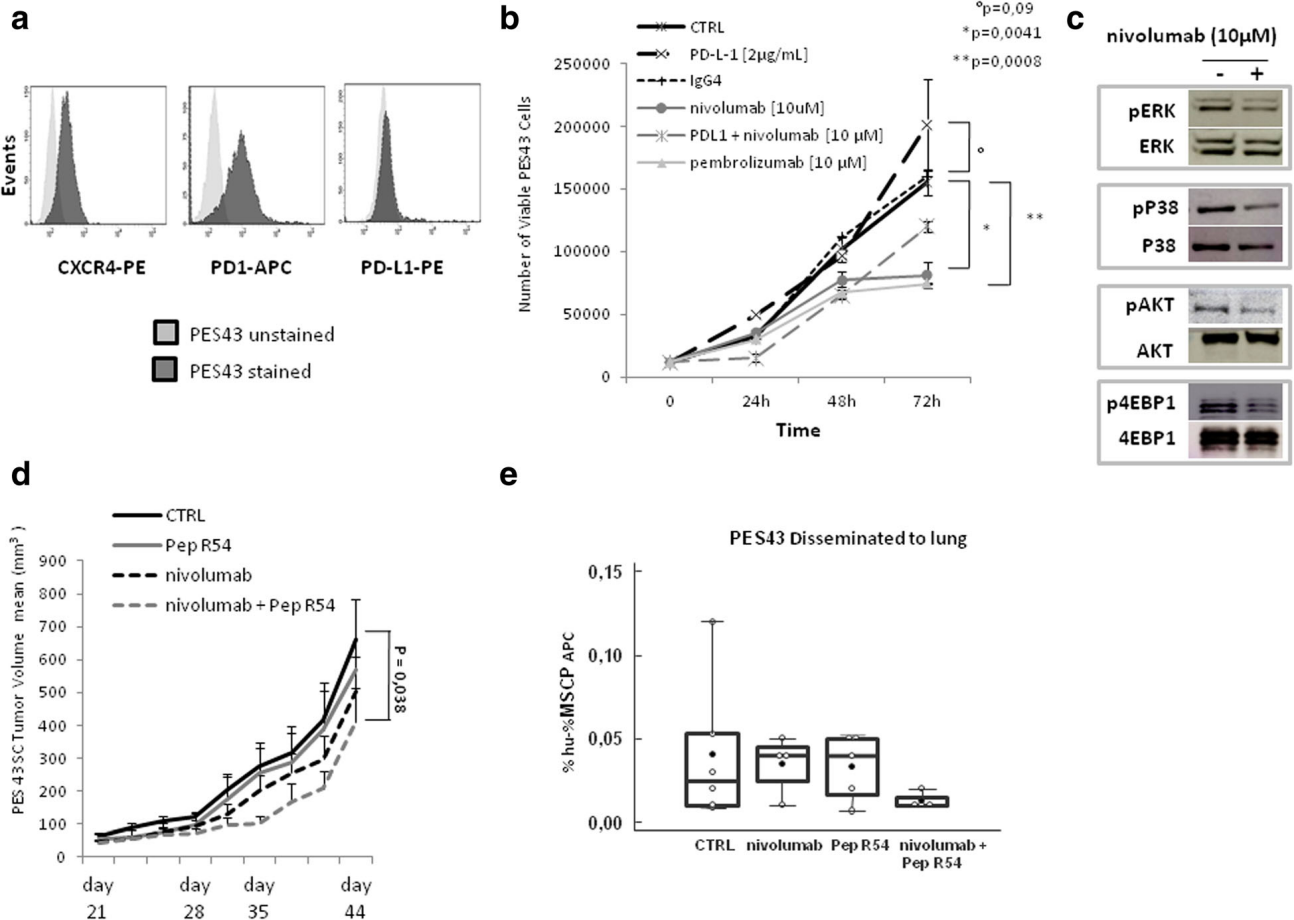
Pep R54 in combination with nivolumab inhibits PES43 human melanoma cell growth, signaling and tumor growth. **a**. PD-1/CD279 (Clone HA2-7B1), PD-L1 (Clone MIH1) and CXCR4 (Clone 12G5) expression in PES43 by flow cytometry. **b**. PES43 cell growth in the presence of nivolumab or pembrolizumab [10 μM], non-specific IgG4[10 μM], PD-L1 [2μg/mL]. Growth curve graph (mean Number of Viable PES43 Cells ± SEM). **c**. Immunoblotting for ERK1/2, P38, AKT, 4EBP1 phosphorylated protein in PES43, PD-L-1 [2 μg/mL] plus nivolumab [10 μM] (6 h incubation); representative data from one of three experiments. **d**. Athymic mice were subcutaneously inoculated with 2.5 × 10^6^ PES43 human melanoma cells and treated for 3 weeks with Peptide R54 (2 mg/kg ip, 5 days per week), nivolumab (5 mg/kg ip, twice weekly) and combination. Tumor volume mm^3^ ± SEM: untreated 622.72 ± 119; nivolumab 503.47 ± 107; Pep R54 567 ± 214; nivolumab + Pep R54 410.33 ± 105). (untreated mice n = 8; nivolumab n = 8; Pep R54 n = 8; nivolumab + Pep R54 *n* = 9); **e.** Lung disseminated cells (DTC) PES43 cells were detected through flow cytometry as % hu- %MSCP APC positive cells (untreated mice n = 6; nivolumab n = 4; Pep R54 n = 5; nivolumab + Pep R54 n = 4) (empty dot = sample; black dot mean)

**Fig. 7:**
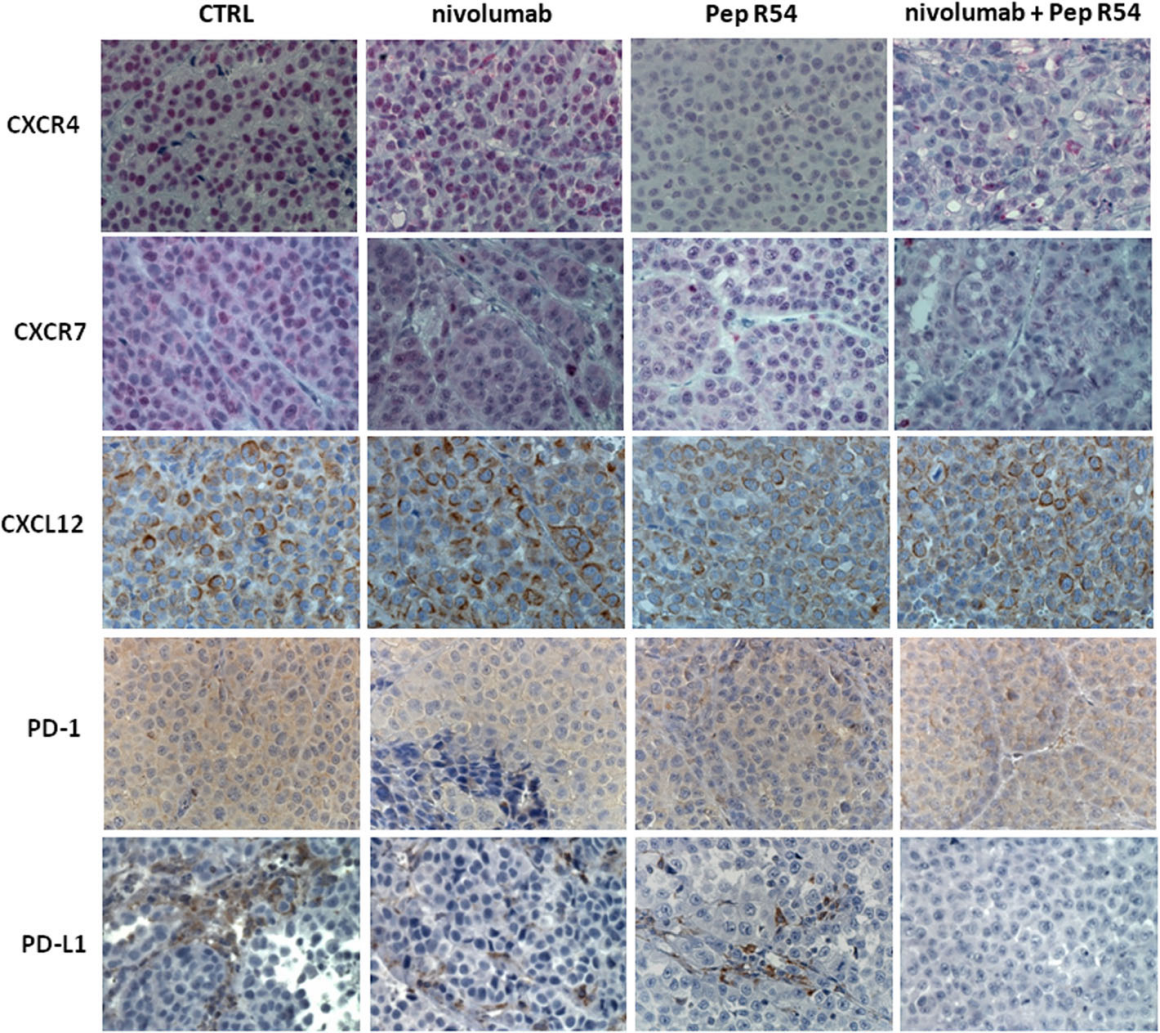
Pep R54 in combination with nivolumab reduced the expression of CXCR4-CXCL12 and PD-L1 in PES43 tumors. Representative IHC pictures (magnification 400x) for CXCR4, CXCR7 (red staining), CXCL12, PD-1 and PD-L1 expression (brown staining) in PES43 collected tumors from mice treated with Pep R54, nivolumab or combined treatment

Recently, intrinsic PD-1 signaling has been described in melanoma, lung, bladder cancer and hepatocellular carcinoma [5]. To dissect the effect of anti-PD-1 and CXCR4 targeting on human cancer cells PES43 human melanoma [25] xenograft was employed. As shown in Fig. 6a PES43 cells express PD-1 (61,3%); low level of PD-L1 (4.1%) and high CXCR4 (44,5%). In vitro human anti-PD-1, nivolumab or pembrolizumab (10 μM), reduced PES43 cells growth (Fig. 6b) and nivolumab (10 μM) inhibited pERK1/2, pP38 MAPK, pAKT and p4EBP (Fig. 6c). In vivo PES43 cells (2.5 × 10^6^) were subcutaneously injected in athymic mice and treated with Pep R54, a newly developed Pep R derivative (AcHN-Arg-Ala-[DCys-Arg- Nal(2′)-His-Pen]- COOH) with improved serum stability and CXCR4 affinity higher than Pep R (IC50 = 20 ± 2 nM) [19]. Pep R54 (2 mg/kg ip, 5 days week/) and human anti-PD-1, nivolumab (5 mg/kg ip, twice weekly), were ip administered for 3 weeks (Additional file 1: Figure S1B). After 3 weeks of combined treatment a significant reduction in tumor growth was revealed (*p* = 0.038) (Fig. 6d). As a corollary, reduction in plasma lactate was detected in Pep R54 + nivolumab treated animals as compared to untreated mice at 44 days post-treatment [36] (Kruskal Wallis test *P* = 0.0209) (Additional file 1: Figure S2). To evaluate the impact of combined treatment on PES43 migrating to lung, disseminated tumor cells (DTC) were detected in fresh lung tissues as melanoma positive MCSP-APC^+^ cells. Although not statistically different, DTC-PES43 cells were reduced in mice lungs treated with combined treatment while no reduction was detected in nivolumab or Pep R54 treated mice (Fig. 6e, Additional file 1: Figure S3A). As showed in Fig. 7 CXCR4 targeting (Pep R54 or Pep R54 + nivolumab) decreased CXCR4 expression, p-ERK and KI67 (Additional file 1: Fig. S3B) while no effect was detected on CXCL12 and cognate receptor CXCR7 expression (Fig. 7). Pep R54 or combination reduced expression of PD-L1 mainly in stromal cells (Fig. 7).

## Discussion

Although immune checkpoint blockade inhibitors (ICI) have shown convincing results in multiple cancers, the therapeutic efficacy is currently limited to 15–30% of treated cancer patients [37]. Herein the new CXCR4 antagonist Pep R strengthen anti-PD-1 efficacy in two murine cancer models, MC38 colon cancer and B16-hCXCR4 melanoma, respectively reported to be immune responsive [21] and immune resistant cancer models [22, 24]. Increase in anti-PD-1 efficacy derived by TME modification potentiating the recruitment of Granzyme B positive and reducing Tregs cells. As previously reported [33, 38], the increase of Granzyme B positive cells in MC38 tumors derived from mice treated with combined treatment, suggests that CXCR4 inhibition favors T effector access to TME. While Peptide R does not significantly increase the number of GRZB positive cells, it improves the anti-PD-1 efficacy toward a more infiltrated TME. CXCR4 inhibition favors T effector access to TME also in a more immune resistant model such as B16-hCXCR4. Pep R also reduced Treg infiltration in MC38 and B16-hCXCR4 tumors rendering the TME more immunoresponsive to anti-PD-1 therapy, as previously reported for B16 melanoma [39]. CXCL12 and PD-L1 expression were reduced by Pep R treatment possibly through impairment of stromal/immuneregulatory cell recruitment [12] and/ or transcriptional regulation [40] while CXCR4 and PD-L1 expression were reduced in tumors treated with anti-PD-1 + Pep R. It was previously demonstrated that CXCR4 antagonists reshape TME favoring access of T effector and reducing the immunoregulatory cells in a model of pancreatic cancer [11], hepatocellular carcinoma [41] colorectal cancer [14] and ovarian cancer [13]. Potentiation in anti-CTLA-4 and anti-PD-1 efficacy was obtained with B16-GM-CSF expressing cell line (GVAX) [35, 39]. Interestingly GM-CSF downregulated both CXCR4 and CXCL12 expression in bone marrow [42]. In human metastatic triple negative breast cancer (TNBC) the dense fibrotic stroma is immunosuppressive and liver and lung metastases tend to be highly fibrotic excluding cytotoxic T lymphocytes (CTLs). Among genes that are associated with stromal T-lymphocyte exclusion there is CXCL12 [43]. In a murine model of murine TNBC, the unique CXCR4 FDA approved antagonist, plerixafor, decreases fibrosis, increases CTL infiltration, and decreases immunosuppression doubling the response to immune checkpoint blockers [43]. CXCR4 is overexpressed on Tregs, mainly the bone marrow retained Tregs, and CXCR4 peptide antagonist impairs Tregs function [34]. Thus dual blockade of CXCL12-CXCR4 and PD-1-PD-L1 synergistically increases Teff/suppressive immune population in murine tumor models. Although not significant the combined treatment modified the content of myeloid-derived suppressor cells (MDSC) and plasmacytoid DC (pDC) in B16-hCXCR4. This can be explained by the TME-produced CXCL12 that is attractive not only for CXCR4+ Treg, but also for MDSC and pDC [44–46]. It was reported that pDCs established an immunosuppressive TME impairing the response to TLR7/9 activation and decreasing IFN-α production [47]. Moreover, genetic deletion of CXCR4 in myeloid cells (CXCR4MyeΔ/Δ) significantly reduced melanoma tumor growth enhancing the NK antitumor immune response. These data suggest that CXCR4-mediated signals from myeloid cells suppress NK cell–mediated tumor surveillance, and thereby enhance tumor growth [48]. In terms of tumor growth, Pep R seems to confer a delay in tumor growth within the first week of treatment while a gain in growth is showed during the second week. Previously published data showed that Pep R reduced the growth of human renal cancer cells SN12C xenograft [15] while Peptide S, although not impairing B16F10 tumor growth, reduced lung metastasis [49]. In U87MG glioblastoma growth was unaffected by CXCR4 antagonists, AMD3100 and Pep R [50]. Although not statistically significant this trend deserve further investigation. Most studies on PD-1 expression have focused on immune cells, rendering its potential expression and functions in tumor cells remaining largely unclear. To investigate the role of intrinsic PD-1 signaling in neoplastic cells, the effect of human anti-PD-1, nivolumab, was evaluated in combination with the newest and most powerful CXCR4 antagonist Pep R54 [20] in a xenograft model of human melanoma. The combined effect of nivolumab + Pep R54 suggests a dual role for CXCR4 antagonism targeting tumoral cells and microenvironment. Herein, combined treatment of nivolumab + Pep R54 impaired tumor growth of human melanoma PES43 cells expressing PD-1 and CXCR4. In athymic mice, double targeting of CXCR4 and PD-1 significantly reduced human melanoma tumor growth as T cells independent effect. Targeting PD-1 and CXCR4 on PES43 melanoma cells reduced cell growth and inhibited survival signaling (pERK/pAkt) [15–17] strengthen the effect of nivolumab that impaired pERK/pAkt and p4EBP1. We hypothesized that the CXCR4 antagonist Pep R54 plus anti-PD-1 simultaneously inhibits two core pathways of tumor proliferation, P-ERK/pAKT and p4EBP1.

## Conclusion

Taken together these results demonstrate that CXCR4 antagonist Peptide R regulates access and function of effector/regulatory cells of the TME creating a rationale for combined therapy with ICI. Pep R potentiated the efficacy of anti-PD-1 through immune cell trafficking manipulation in two syngeneic models with different immunogenicity. In addition, the CXCR4 antagonist Peptide R54 potentiates the inhibition of cell-intrinsic PD-1, T cell-independent response, in human melanoma xenograft providing relevant information for combinatorial approaches to enhance antitumor immunity.

## Supplementary information

**Additional file 1: Figure S1.** In vivo Scheme of treatment. **A.** Scheme of treatment for syngeneic murine models: Peptide R was intraperitoneally administered 5 day/week for 2, Anti-PD-1 was intraperitoneally administered at Day 1–4–7-10-13. **B.** Scheme of treatment for human PES43 melanoma xenograft: Peptide R54 was intraperitoneally administered 5 day/week for 3 weeks, Anti-PD-1 nivolumab was intraperitoneally administered at Day 1–4–7-10-13-16-19. **Figure S2.** CXCR4 antagonist Pep R54 in combination with nivolumab reduced plasma lactate. Reduction in plasma lactate was revealed in Pep R54 + nivolumab treated animals as compared to untreated mice at 44 days post-treatment. Lactate dehydrogenase (LDH) blood level from retro*-*orbital plexus (100 μl) blood sampling (plasma concentration mg/dL, mean ± SD). **Figure S3. A.** Peptide R54 in combination with nivolumalb reduced PES43 lung nodules. Representative PES43 metastasis in athymic mouse lung. Metastatic nodules were evaluated 8 weeks after PES43 subcutaneous injection (3/5 untreated mice, 1/4 nivolumab, 1/6 Pep R54, and 0/4 Pep R54 + nivolumab treated mice). Upper: H&E of lungs from PES43 xenograft mice: Lower: IHC for MELAN-MART1. **B.** Peptide R54 in combination with nivolumab reduced CXCR4-, P-ERK downstream signaling and Ki67 in PES43 xenograft. **B.** Representative IHC pictures (magnification 400x) for P-ERK downstream signaling pathway and Ki67 with membrane/cytoplasmic and nuclear localization respectively.

## Data Availability

The datasets used and/or analyzed during the current study are available from the corresponding author on reasonable request.

## Abbreviations

3Rs: Replacement, Reduction and Refinement
CTLA-4: Cytotoxic T lymphocyte–associated protein 4
CTLs: Cytotoxic T lymphocytes
DTC: Disseminated tumor cells
FACS: Fluorescence-activated cell sorting
GRZB: Granzyme B
H&E: Hematoxylin and eosin stain
HIER: Heat-induced epitope retrieval
ICI: Immune checkpoints inhibition
IHC: Immunohistochemistry
MCSP: Melanoma-associated chondroitin sulfate proteoglycan
PD-1: Programmed cell death 1
pDCs: Plasmacytoid Dendritic cells
Pep R: Peptide R
Pep R54: Peptide R54
RMANOVA: Repeated measures analysis of variance
RTV: Relative Tumor volume
TME: Tumor microenvironment
Tregs: Regulatory T cells

## References

[1] Ribas A, Wolchok JD (2018). Cancer immunotherapy using checkpoint blockade. Science (New York, NY).

[2] Garcia-Diaz A, Shin DS, Moreno BH, Saco J, Escuin-Ordinas H, Rodriguez GA (2017). Interferon Receptor Signaling Pathways Regulating PD-L1 and PD-L2 Expression. Cell reports..

[3] Sharma P, Hu-Lieskovan S, Wargo JA, Ribas A (2017). Primary, Adaptive, and Acquired Resistance to Cancer Immunotherapy. Cell..

[4] Li H, Li X, Liu S, Guo L, Zhang B, Zhang J (2017). Programmed cell death-1 (PD-1) checkpoint blockade in combination with a mammalian target of rapamycin inhibitor restrains hepatocellular carcinoma growth induced by hepatoma cell-intrinsic PD-1. Hepatology (Baltimore, Md).

[5] Kleffel S, Posch C, Barthel SR, Mueller H, Schlapbach C, Guenova E (2015). Melanoma Cell-Intrinsic PD-1 Receptor Functions Promote Tumor Growth. Cell..

[6] Zhang D, Sun X, Gupta HB, Reyes RM, Svatek RS, Curiel TJ (2018). Cell-intrinsic PD-L1 and PD-1 signal effects in bladder cancer. The Journal of Immunology..

[7] Yao H, Wang H, Li C, Fang JY, Xu J (2018). Cancer Cell-Intrinsic PD-1 and Implications in Combinatorial Immunotherapy. Frontiers in immunology..

[8] Scala S (2015). Molecular Pathways: Targeting the CXCR4-CXCL12 Axis--Untapped Potential in the Tumor Microenvironment. Clinical Cancer Research: an official journal of the American Association for Cancer Research..

[9] Chen Y, Ramjiawan RR, Reiberger T, Ng MR, Hato T, Huang Y (2014). CXCR4 inhibition in tumor microenvironment facilitates anti-PD-1 immunotherapy in sorafenib-treated HCC in mice. Hepatology..

[10] Righi E, Kashiwagi S, Yuan J, Santosuosso M, Leblanc P, Ingraham R (2011). CXCL12/CXCR4 blockade induces multimodal antitumor effects that prolong survival in an immunocompetent mouse model of ovarian cancer. Cancer research..

[11] Feig C, Jones JO, Kraman M, Wells RJ, Deonarine A, Chan DS (2013). Targeting CXCL12 from FAP-expressing carcinoma-associated fibroblasts synergizes with anti-PD-L1 immunotherapy in pancreatic cancer. Proceedings of the National Academy of Sciences of the United States of America..

[12] Joyce JA, Fearon DT (2015). T cell exclusion, immune privilege, and the tumor microenvironment. Science (New York, NY).

[13] Zeng Y, Li B, Liang Y, Reeves PM, Qu X, Ran C (2019). Dual blockade of CXCL12-CXCR4 and PD-1-PD-L1 pathways prolongs survival of ovarian tumor-bearing mice by prevention of immunosuppression in the tumor microenvironment. FASEB journal : official publication of the Federation of American Societies for Experimental Biology..

[14] Zboralski D, Hoehlig K, Eulberg D, Fromming A, Vater A (2017). Increasing Tumor-Infiltrating T Cells through Inhibition of CXCL12 with NOX-A12 Synergizes with PD-1 Blockade. Cancer immunology research..

[15] Portella L, Vitale R, De Luca S, D'Alterio C, Ierano C, Napolitano M (2013). Preclinical development of a novel class of CXCR4 antagonist impairing solid tumors growth and metastases. PloS one..

[16] Fontanella R, Pelagalli A, Nardelli A, D'Alterio C, Ierano C, Cerchia L (2016). A novel antagonist of CXCR4 prevents bone marrow-derived mesenchymal stem cell-mediated osteosarcoma and hepatocellular carcinoma cell migration and invasion. Cancer letters..

[17] Santagata S, Portella L, Napolitano M, Greco A, D’Alterio C, Barone MV (2017). A novel CXCR4-targeted near-infrared (NIR) fluorescent probe (Peptide R-NIR750) specifically detects CXCR4 expressing tumors. Scientific Reports..

[18] Ierano C, Portella L, Lusa S, Salzano G, D'Alterio C, Napolitano M (2016). CXCR4-antagonist Peptide R-liposomes for combined therapy against lung metastasis. Nanoscale..

[19] Di Maro S, Di Leva FS, Trotta AM, Brancaccio D, Portella L, Aurilio M (2017). Structure-Activity Relationships and Biological Characterization of a Novel, Potent, and Serum Stable C-X-C Chemokine Receptor Type 4 (CXCR4) Antagonist. Journal of Medicinal Chemistry..

[20] Di Maro S, Trotta AM, Brancaccio D, Di Leva FS, La Pietra V, Ierano C (2016). Exploring the N-Terminal Region of C-X-C Motif Chemokine 12 (CXCL12): Identification of Plasma-Stable Cyclic Peptides As Novel, Potent C-X-C Chemokine Receptor Type 4 (CXCR4) Antagonists. Journal of Medicinal Chemistry..

[21] Ngiow SF, Young A, Jacquelot N, Yamazaki T, Enot D, Zitvogel L (2015). A Threshold Level of Intratumor CD8+ T-cell PD1 Expression Dictates Therapeutic Response to Anti-PD1. Cancer Res..

[22] Moreno BH, Zaretsky JM, Garcia-Diaz A, Tsoi J, Parisi G, Robert L (2016). Response to programmed cell death-1 blockade in a murine melanoma syngeneic model requires costimulation, CD4, and CD8 T cells. Cancer immunology research..

[23] Cross RS, Malaterre J, Davenport AJ, Carpinteri S, Anderson RL, Darcy PK (2015). Therapeutic DNA vaccination against colorectal cancer by targeting the MYB oncoprotein. Clinical & translational immunology..

[24] Kuczynski EA, Krueger J, Chow A, Xu P, Man S, Sundaravadanam Y (2018). Impact of Chemical-Induced Mutational Load Increase on Immune Checkpoint Therapy in Poorly Responsive Murine Tumors. Molecular cancer therapeutics..

[25] Scala S, Giuliano P, Ascierto PA, Ierano C, Franco R, Napolitano M (2006). Human melanoma metastases express functional CXCR4. Clinical Cancer Research: an official journal of the American Association for Cancer Research..

[26] Zheng W, Skowron KB, Namm JP, Burnette B, Fernandez C, Arina A (2016). Combination of radiotherapy and vaccination overcomes checkpoint blockade resistance. Oncotarget..

[27] Wu J (2010). Statistical inference for tumor growth inhibition T/C ratio. Journal of Biopharmaceutical Statistics..

[28] Mosely SI, Prime JE, Sainson RC, Koopmann JO, Wang DY, Greenawalt DM (2017). Rational Selection of Syngeneic Preclinical Tumor Models for Immunotherapeutic Drug Discovery. Cancer Immunology Research..

[29] Grasselly C, Denis M, Bourguignon A, Talhi N, Mathe D, Tourette A (2018). The Antitumor Activity of Combinations of Cytotoxic Chemotherapy and Immune Checkpoint Inhibitors Is Model-Dependent. Frontiers in Immunology.

[30] Cubas R, Moskalenko M, Cheung J, Yang M, McNamara E, Xiong H, et al. Chemotherapy Combines Effectively with Anti–PD-L1 Treatment and Can Augment Antitumor Responses. The Journal of Immunology. 2018;ji1800275. 10.4049/jimmunol.1800275 30209192

[31] Chen S, Lee L-F, Fisher TS, Jessen B, Elliott M, Evering W (2015). Combination of 4-1BB Agonist and PD-1 Antagonist Promotes Antitumor Effector/Memory CD8 T Cells in a Poorly Immunogenic Tumor Model. Cancer immunology research..

[32] Daneshmandi S, Wegiel B, Seth P. Blockade of Lactate Dehydrogenase-A (LDH-A) Improves Efficacy of Anti-Programmed Cell Death-1 (PD-1) Therapy in Melanoma. Cancers. 2019;11(4). 10.3390/cancers11040450 PMC6521327 30934955

[33] Bezman NA, Jhatakia A, Kearney AY, Brender T, Maurer M, Henning K (2017). PD-1 blockade enhances elotuzumab efficacy in mouse tumor models. Blood advances..

[34] Santagata S, Napolitano M, D'Alterio C, Desicato S, Maro SD, Marinelli L (2017). Targeting CXCR4 reverts the suppressive activity of T-regulatory cells in renal cancer. Oncotarget..

[35] Quezada SA, Peggs KS, Curran MA, Allison JP (2006). CTLA4 blockade and GM-CSF combination immunotherapy alters the intratumor balance of effector and regulatory T cells. The Journal of Clinical Investigation..

[36] Kouidhi S, Ben Ayed F, Benammar EA (2018). Targeting Tumor Metabolism: A New Challenge to Improve Immunotherapy. Frontiers in Immunology..

[37] Kamada T, Togashi Y, Tay C, Ha D, Sasaki A, Nakamura Y (2019). PD-1(+) regulatory T cells amplified by PD-1 blockade promote hyperprogression of cancer. Proceedings of the National Academy of Sciences of the United States of America..

[38] Larimer BM, Wehrenberg-Klee E, Dubois F, Mehta A, Kalomeris T, Flaherty K (2017). Granzyme B PET Imaging as a Predictive Biomarker of Immunotherapy Response. Cancer Res..

[39] Curran MA, Montalvo W, Yagita H, Allison JP (2010). PD-1 and CTLA-4 combination blockade expands infiltrating T cells and reduces regulatory T and myeloid cells within B16 melanoma tumors. Proceedings of the National Academy of Sciences..

[40] Jung YD, Shim JW, Park SJ, Choi SH, Yang K, Heo K (2015). Downregulation of UHRF1 promotes EMT via inducing CXCR4 in human cancer cells. International journal of oncology..

[41] Chen Y, Ramjiawan RR, Reiberger T, Ng MR, Hato T, Huang Y (2015). CXCR4 inhibition in tumor microenvironment facilitates anti-programmed death receptor-1 immunotherapy in sorafenib-treated hepatocellular carcinoma in mice. Hepatology (Baltimore, Md).

[42] Rosales C. Neutrophil: A Cell with Many Roles in Inflammation or Several Cell Types? Frontiers in Physiology. 2018;9(113). 10.3389/fphys.2018.00113 PMC5826082 29515456

[43] Chen IX, Chauhan VP, Posada J, Ng MR, Wu MW, Adstamongkonkul P, et al. Blocking CXCR4 alleviates desmoplasia, increases T-lymphocyte infiltration, and improves immunotherapy in metastatic breast cancer. Proceedings of the National Academy of Sciences. 2019;201815515. 10.1073/pnas.1815515116 PMC6410779 30700545

[44] Gabrilovich DI, Nagaraj S (2009). Myeloid-derived suppressor cells as regulators of the immune system. Nature Reviews Immunology..

[45] Katoh H, Hosono K, Ito Y, Suzuki T, Ogawa Y, Kubo H (2010). COX-2 and prostaglandin EP3/EP4 signaling regulate the tumor stromal proangiogenic microenvironment via CXCL12-CXCR4 chemokine systems. The American journal of pathology..

[46] Gadalla R, Hassan H, Ibrahim SA, Abdullah MS, Gaballah A, Greve B, et al. Tumor microenvironmental plasmacytoid dendritic cells contribute to breast cancer lymph node metastasis via CXCR4/SDF-1 axis. Breast Cancer Research and Treatment. 2019. 10.1007/s10549-019-05129-8 30632021

[47] Demoulin S, Herfs M, Delvenne P, Hubert P (2013). Tumor microenvironment converts plasmacytoid dendritic cells into immunosuppressive/tolerogenic cells: insight into the molecular mechanisms. Journal of Leukocyte Biology..

[48] Yang JM, Kumar A, Vilgelm AE, Chen SC, Ayers GD, Novitskiy SV (2018). Loss of CXCR4 in Myeloid Cells Enhances Antitumor Immunity and Reduces Melanoma Growth through NK Cell and FASL Mechanisms. Cancer Immunology Research..

[49] Mei L, Liu Y, Zhang Q, Gao H, Zhang Z, He Q (2014). Enhanced antitumor and anti-metastasis efficiency via combined treatment with CXCR4 antagonist and liposomal doxorubicin. Journal of Controlled Release: official journal of the Controlled Release Society..

[50] Mercurio L, Ajmone-Cat MA, Cecchetti S, Ricci A, Bozzuto G, Molinari A (2016). Targeting CXCR4 by a selective peptide antagonist modulates tumor microenvironment and microglia reactivity in a human glioblastoma model. J Exp Clin Cancer Res..

