# Supplementary figures and images for: Targeting CXCR4 potentiates anti-PD-1 efficacy modifying the tumor microenvironment and inhibiting neoplastic PD-1
Source: J Exp Clin Cancer Res. 2019 Oct 28;38:432. doi: 10.1186/s13046-019-1420-8 (PMC6819555; doi:10.1186/s13046-019-1420-8)

## Supplementary Figure 1

### Scheme of treatment

A

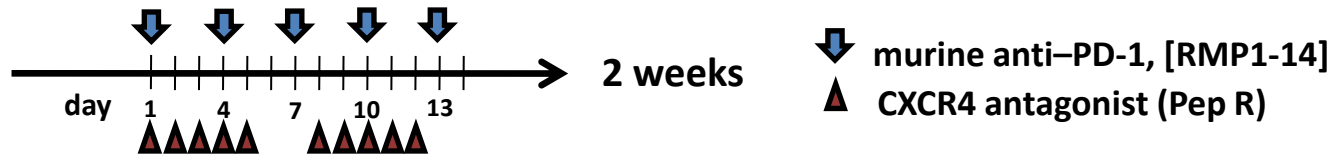

B

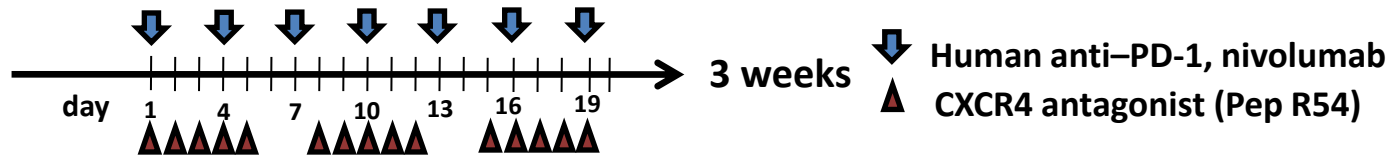

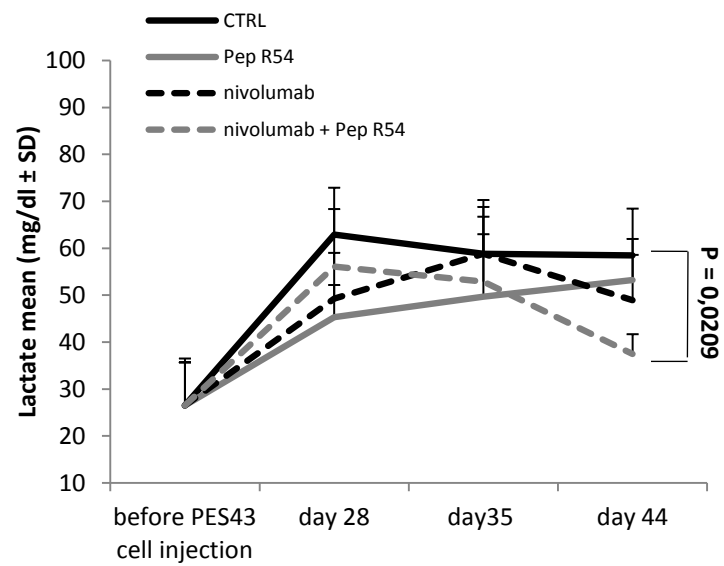

**A**

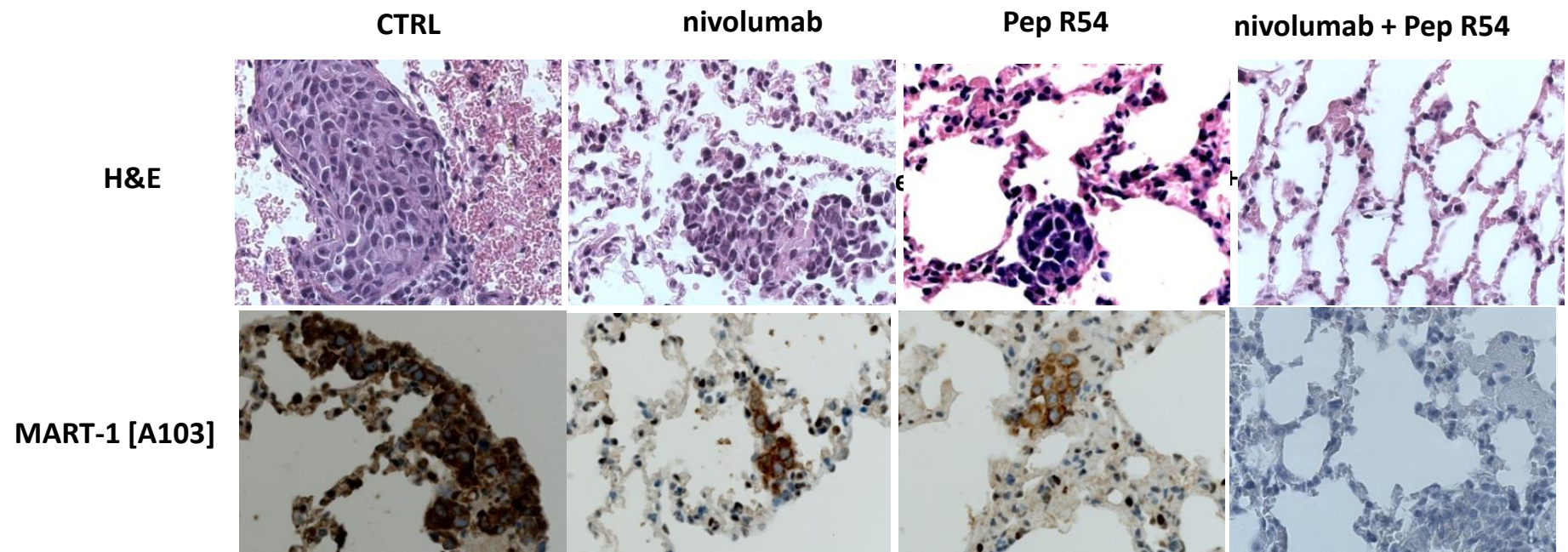

**B**

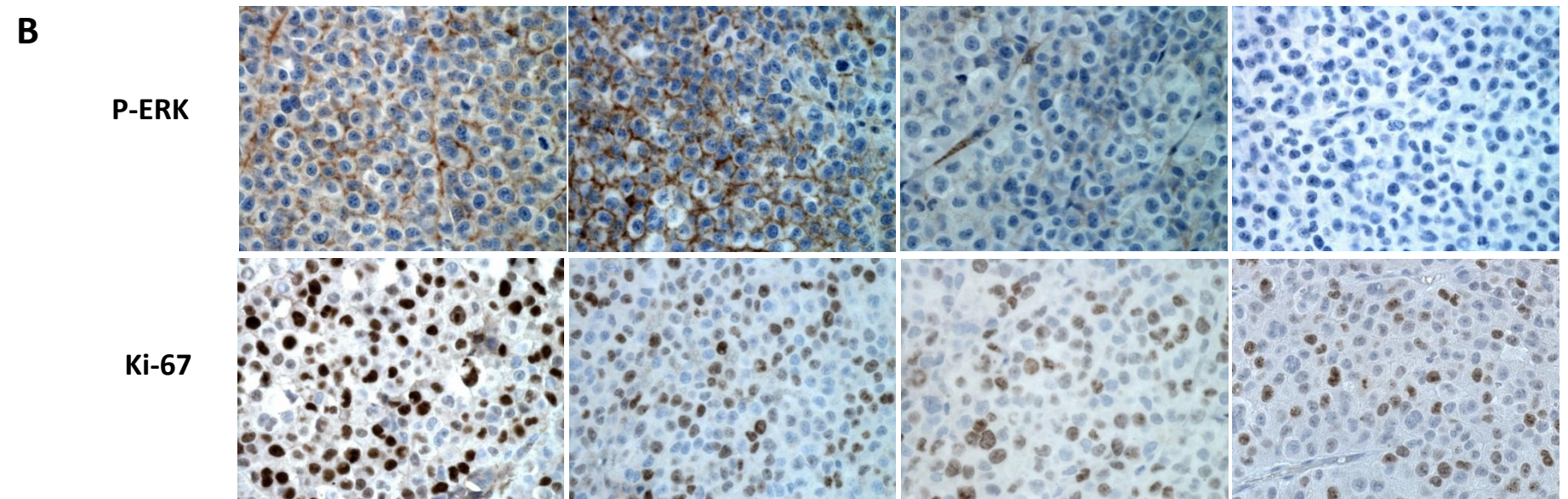

Supplement: Supplementary file 1 — Additional file 1: Figure S1. In vivo Scheme of treatment. A. Scheme of treatment for syngeneic murine models: Peptide R was intraperitoneally administered 5 day/week for 2, Anti-PD-1 was intraperitoneally administered at Day 1–4–7-10-13. B. Scheme of treatment for human PES43 melanoma xenograft: Peptide R54 was intraperitoneally administered 5 day/week for 3 weeks, Anti-PD-1 nivolumab was intraperitoneally administered at Day 1–4–7-10-13-16-19. Figure S2. CXCR4 antagonist Pep R54 in combination with nivolumab reduced plasma lactate. Reduction in plasma lactate was revealed in Pep R54 + nivolumab treated animals as compared to untreated mice at 44 days post-treatment. Lactate dehydrogenase (LDH) blood level from retro-orbital plexus (100 μl) blood sampling (plasma concentration mg/dL, mean ± SD). Figure S3. A. Peptide R54 in combination with nivolumalb reduced PES43 lung nodules. Representative PES43 metastasis in athymic mouse lung. Metastatic nodules were evaluated 8 weeks after PES43 subcutaneous injection (3/5 untreated mice, 1/4 nivolumab, 1/6 Pep R54, and 0/4 Pep R54 + nivolumab treated mice). Upper: H&E of lungs from PES43 xenograft mice: Lower: IHC for MELAN-MART1. B. Peptide R54 in combination with nivolumab reduced CXCR4-, P-ERK downstream signaling and Ki67 in PES43 xenograft. B. Representative IHC pictures (magnification 400x) for P-ERK downstream signaling pathway and Ki67 with membrane/cytoplasmic and nuclear localization respectively. [file 13046_2019_1420_MOESM1_ESM.pdf]
